# Cognitive Collaborations: Bidirectional Functional Connectivity Between the Cerebellum and the Hippocampus

**DOI:** 10.3389/fnsys.2015.00177

**Published:** 2015-12-22

**Authors:** Wilson Yu, Esther Krook-Magnuson

**Affiliations:** Department of Neuroscience, University of MinnesotaMinneapolis, MN, USA

**Keywords:** cerebellum, hippocampus, connectivity, spatial, temporal, epilepsy

## Abstract

There is a growing recognition that the utility of the cerebellum is not limited to motor control. This review focuses on the particularly novel area of hippocampal-cerebellar interactions. Recent work has illustrated that the hippocampus and cerebellum are functionally connected in a bidirectional manner such that the cerebellum can influence hippocampal activity and vice versa. This functional connectivity has important implications for physiology, including spatial navigation and timing-dependent tasks, as well as pathophysiology, including seizures. Moving forward, an improved understanding of the critical biological underpinnings of these cognitive collaborations may improve interventions for neurological disorders such as epilepsy.

## Introduction

The cerebellum has traditionally been associated with motor control, motor learning, and coordination (Glickstein, 2007; Manto et al., 2012), but it is now often understood to be more broadly involved in cognitive functions as well (Strick et al., 2009; Popa et al., 2014; Taylor and Ivry, 2014). This is supported in part anatomically by the marked expansion of the human cerebellum (Matano, 2001) and the connections it has developed with areas supporting cognition (Leiner et al., 1986, 1989; Weaver, 2005). Notably, recent studies have reported important functional interactions between the cerebellum and the hippocampal formation (Rochefort et al., 2011; Krook-Magnuson et al., 2014; Onuki et al., 2015). For instance, while the process of pattern separation has been studied primarily within subregions of the hippocampal formation, it was recently reported that the cerebellum is also actively engaged during pattern separation tasks (Paleja et al., 2014). Conversely, tasks known to be heavily cerebellum-dependent can be significantly influenced by the hippocampus (Hoffmann and Berry, 2009; Wikgren et al., 2010; Hoffmann et al., 2015). A recognition of cerebellar-hippocampal interactions represents a paradigm shift as it challenges us to re-examine traditional notions of spatial and temporal processing and to better appreciate the collaborative efforts involved. Additionally, the broader role of the cerebellum beyond motor control has critical implications for understanding neurological disorders, including autism and epilepsy. Hippocampal-cerebellar interactions in particular may be especially relevant to temporal lobe epilepsy, as discussed later in this review.

## Cerebellar-hippocampal connectivity: many paths of potential influence

**Figure 1:**
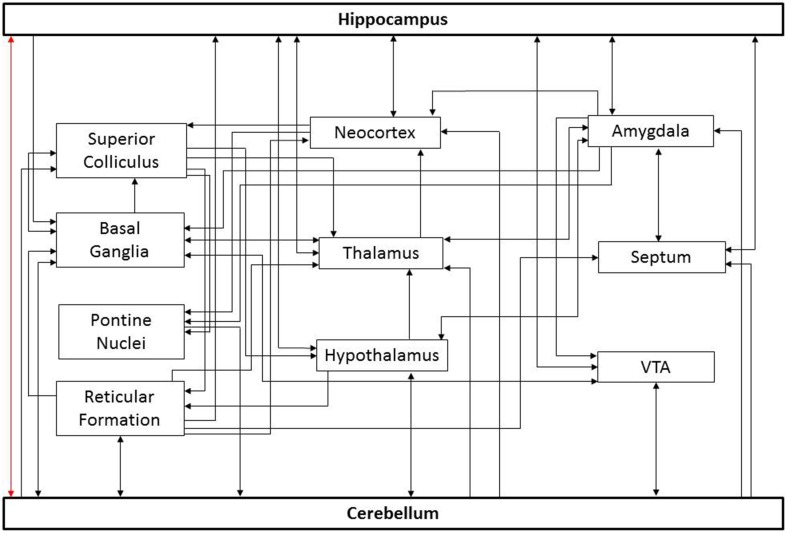
**Potential paths of influence**. This simplified diagram illustrates potential pathways underlying cerebello-hippocampal functional connectivity. For the sake of simplicity, only general connections between structures are shown; important subdivisions of depicted structures have been omitted. Similarly, only a subset of potential routes is depicted. As noted in the text, there is controversy surrounding a potential direct connection between these structures (red arrow). Additional connections depicted include input to the cerebellum from the basal ganglia (Bostan and Strick, 2010), reticular formation (Pierce et al., 1977; Verveer et al., 1997; Luo and Sugihara, 2014), pontine nuclei (Kawamura and Hashikawa, 1981), hypothalamus (Dietrichs and Haines, 1984, 1986, 1989; Onat and Cavdar, 2003), and ventral tegmental area (VTA) (Snider et al., 1976; Oades and Halliday, 1987); projections from the cerebellum to the superior colliculus (Person et al., 1986a), basal ganglia (Bostan and Strick, 2010), reticular formation (Elisevich et al., 1985; Person et al., 1986b; Perciavalle et al., 1989; Teune et al., 2000; Almeida et al., 2002), hypothalamus (Dietrichs and Haines, 1989; Onat and Cavdar, 2003), thalamus (Haroian et al., 1981; Asanuma et al., 1983; Angaut et al., 1985; Person et al., 1986a), neocortex (Harper and Heath, 1973; Clower et al., 2005), VTA (Oades and Halliday, 1987; Snider et al., 1976), septum (Paul et al., 1973; Heath et al., 1978), and amygdala (Heath and Harper, 1974); projections to the hippocampus from the neocortex (Canto et al., 2008; Ohara et al., 2013), septum (Ohara et al., 2013), reticular formation (Lewis and Shute, 1967; Köhler and Steinbusch, 1982; Andersen et al., 1983), hypothalamus (Lima et al., 2013; Soussi et al., 2015), thalamus (Vertes, 2015), VTA (Kahn and Shohamy, 2013) and amygdala (French et al., 2003); projections from the hippocampus to the amygdala (Ishikawa and Nakamura, 2006), basal ganglia (Floresco et al., 2001), hypothalamus (Swanson and Cowan, 1977), thalamus (Swanson and Cowan, 1977), neocortex (Swanson and Cowan, 1977), VTA (Kahn and Shohamy, 2013), and septum (Swanson and Cowan, 1977). Similarly, connections exist between these intermediate structures which may indirectly influence the cerebellum and hippocampus.

The relevant connectivity between the cerebellum and hippocampus is not fully understood, in part because there are several potential pathways connecting these structures (Figure 1). The simplest route would be a direct connection. However, the existence of such a route is controversial. Electrical stimulation of the cerebellum can evoke potentials in the hippocampus (reported for cat, rat, and rhesus monkey; Heath and Harper, 1974; Snider and Maiti, 1976; Heath et al., 1978; Newman and Reza, 1979). An anatomical study in cats and rhesus monkey found degenerating fibers in the hippocampus after a lesion of the cerebellar fastigial nucleus (Heath and Harper, 1974). In chicken, lesion and retrograde tracing studies also suggest a direct pathway from the hippocampal formation to the cerebellum (Liu et al., 2012). Finally, in humans, a direct white matter bundle connecting the hippocampus and cerebellum was recently reported using probabilistic Constrained Spherical Deconvolution tractography (Arrigo et al., 2014). These studies indicate that these structures might have a direct anatomical substrate by which to influence one another.

However, it is important to recognize that the bidirectional influence of the cerebellum and hippocampus on one another need not rely on a direct monosynaptic connection. Once indirect pathways are considered, there are many potential routes connecting these structures (Figure 1). Optogenetic techniques provide the opportunity to selectively manipulate cell types and pathways with temporal precision, and may allow identification of key routes in cerebellar-hippocampal interactions in the future (Boyden et al., 2005; Deisseroth, 2011; Armstrong et al., 2013; Krook-Magnuson and Soltesz, 2015; Krook-Magnuson et al., 2015b). Identifying which of these potential pathways actually play a critical role in the functional connectivity will aid in understanding information processing and may provide additional structures or pathways to target for treating neurological disorders. While there is currently limited data on the pathways mediating cerebellar-hippocampal interactions, it is becoming increasingly evident that they are functionally significant.

## Cerebellar-hippocampal interactions in spatial processing

One of the key functions of the hippocampus is spatial navigation (Eichenbaum, 2000; Dumont and Taube, 2015). However, it does not perform this role in isolation, but rather relies on information input from other brain areas. Studies have demonstrated an important contribution of the cerebellum in the formation of spatial representations (Lalonde and Botez, 1990; Wallesch and Horn, 1990; Petrosini et al., 1998). Spatial navigation involves a combination of internal cues such as proprioceptive and vestibular input, as well as external cues such as landmarks (Dumont and Taube, 2015). The cerebellum receives input from the vestibular nucleus (Hitier et al., 2014) and is believed to play a crucial role in encoding inertial motion and transforming self-motion vestibular information from an egocentric head-centered reference into allocentric Earth-referenced spatial orientation (Yakusheva et al., 2007; Angelaki et al., 2010). Transgenic mice with impaired cerebellar function have deficits in goal-directed spatial trajectories (Burguière et al., 2005), retention of spatial memory (Hilber et al., 1998), and tasks requiring use of self-motion information (Rochefort et al., 2011). The functional connection between the cerebellum and hippocampus in the context of spatial navigation is perhaps most strikingly seen in recordings from hippocampal neurons: animals with certain impairments in cerebellar function have fewer hippocampal place cells, and, when forced to rely on self-motion cues for spatial navigation, place cells show decreased firing rates and reduced stability (Rochefort et al., 2011). Similarly, in healthy animals, removal of vestibular self-motion cues reduces the number of place cells (Ravassard et al., 2013).

Evidence that the cerebellum is not only important for spatial navigation in rodents, but also in humans, comes from a recent human imaging study which demonstrated functional co-activation of the cerebellum and hippocampus during spatial navigation (Igloi et al., 2014). Interestingly, co-activation was found for both allocentric and egocentric navigation tasks, but with hemispheric specialization. The right cerebellum and left hippocampus displayed coactivation during egocentric navigation, while the left cerebellum and right hippocampus displayed coactivation during allocentric navigation. When both egocentric and allocentric navigation occurred in parallel, both circuits were active. This suggests hemispheric specialization and a potential role for the cerebellum in both egocentric and allocentric navigation.

How does the cerebellum influence hippocampal spatial navigation? One proposal is that the cerebellum provides self-motion related information to grid cells in the entorhinal cortex (Passot et al., 2012; Rochefort et al., 2013) and thereby contributes to the formation of spatial representations in the hippocampus (McNaughton et al., 2006). However, given the sensitivity of place cells to cerebellar disturbances (Rochefort et al., 2011), and that place cells can exist in the absence of entorhinal cortex grid cells (Bush and Burgess, 2014; Hales et al., 2014), an alternative or additional mechanism seems likely, and there are many possibilities. For example, as head direction cells are able to maintain their firing properties in the dark (at least in animals with a functional cerebellum), they are assumed to receive self-motion cues (Taube, 2007; Bush and Burgess, 2014), and therefore may be influenced by cerebellar function. However, this is only one potential mechanism and further work is needed to determine which of the many possible pathways (Figure 1) are critically involved in cerebellar contributions to spatial processing in the hippocampus.

## Cerebellar-hippocampal interactions in temporal processing

Just as the cerebellum becomes critical to hippocampal functioning on a hippocampal-dependent task (spatial navigation) when the task requires integration of self-motion cues (a cerebellar forte), the hippocampus can become a vital structure in a cerebellar-dependent task. Specifically, it is well established that the cerebellum is a key structure in conditioned eyeblink responses (Mauk and Thompson, 1987; Moyer et al., 1990; Thompson and Krupa, 1994; Gruart and Yeo, 1995; Kirsch et al., 2003; De Zeeuw and Yeo, 2005; Thompson and Steinmetz, 2009; Longley and Yeo, 2014). Critically, however, when an interval is introduced between the end of the conditioned stimulus and the unconditioned stimulus (i.e., trace eyeblink conditioning), the task (in particular learning of the task, Kim et al., 1995; Takehara et al., 2002, 2003) additionally requires the hippocampus; hippocampal lesions impair the acquisition of trace conditioning responses (Clark et al., 1984; Solomon et al., 1986; Moyer et al., 1990; Clark and Squire, 1998; Ryou et al., 1998; Weiss et al., 1999; Weiss and Disterhoft, 2015). Notably, while the hippocampus plays a central role in spatial navigation, it is also relevant to the coding of time (Eichenbaum, 2014) and, more broadly, declarative and episodic memory (Eichenbaum, 2001; Burgess et al., 2002)—these may be critical functions supplied by the hippocampus to the cerebellum in this task. Despite the cerebellum's hypothesized role in working memory (Kuper et al., 2015), without hippocampal support, the cerebellum appears unable to keep information about the conditioned stimulus “on-line” during the gap between stimuli. Cerebellar cell firing decreases after ~300 ms (Ito, 1982), and the hippocampus may bridge the gap between the conditioned and unconditioned stimulus for the cerebellum. Hippocampal cells have been shown to bridge temporal gaps in other contexts as well, such as during an odor-object pairing task (MacDonald et al., 2011). As in spatial navigation, the dual hippocampal cerebellar dependency of this task has also been demonstrated in humans (Logan and Grafton, 1995; McGlinchey-Berroth et al., 1997). Related to the hippocampus' role in episodic and declarative memory, human studies have further examined the potential need for hippocampal-dependent awareness in the acquisition of trace conditioning (Clark et al., 2002).

It has been proposed that the hippocampal influence on cerebellar function in trace eyeblink conditioning is routed through the medial prefrontal cortex (Figure 1; Weiss and Disterhoft, 2011). However, as dependency on the hippocampus and medial prefrontal cortex show distinct temporal aspects (with the hippocampus rather than the prefrontal cortex being required during learning, Takehara et al., 2003), an alternate route is likely to participate. Interestingly, not only do lesions of the hippocampus disrupt performance on this task (Berry and Thompson, 1979; Solomon et al., 1983), but lesions or inactivation of the cerebellum also disrupts hippocampal responses (Clark et al., 1984; Ryou et al., 1998). This further emphasizes the *bidirectional* functional connectivity between these two structures, although again, the pathway involved remains unresolved.

The cerebellar interpositus nucleus is crucial in eyeblink conditioning (lesions to this nucleus abolishes conditioned hippocampal responses) and projects conditioned response-related activity to the red nucleus and the ventral lateral nucleus of the thalamus (Sears et al., 1996). However, lesions to these downstream areas do not affect conditioning-related activity in the hippocampus, indicating additional pathways are likely involved (Clark et al., 1984; Sears and Steinmetz, 1990; Ryou et al., 1998).

A functional coupling between the hippocampus and cerebellum can additionally be seen in the synchronization of oscillations during eyeblink conditioning, as cerebellar and hippocampal type II theta (Bland, 1986) are synchronized during this task (Singer, 1999; Fries, 2005). Strikingly, presentation of a conditioned stimulus will induce theta when an animal is in a non-theta state, induce a phase reset of theta when presented during spontaneous theta, and increase the degree of hippocampal-cerebellar theta synchrony (McCartney et al., 2004; Hoffmann and Berry, 2009; Wikgren et al., 2010; Nokia et al., 2012). Interestingly, in rabbits and humans alike, scopolamine, a competitive muscarinic acetylcholine receptor antagonist known to block type II theta oscillations (Bland, 1986; Buzsaki, 2002), also inhibits eyeblink conditioning (Salvatierra and Berry, 1989; Solomon et al., 1993). Just as elucidating the relevant pathways connecting the hippocampus and the cerebellum is an area for active research, the mechanism behind this theta reset and synchrony across these structures is currently unknown, and may be mediated by a structure which projects to both the hippocampus and cerebellum, rather than indicating a direct cross-talk *per se*. Potential candidates include the medial septum and the supramammillary nucleus (Figure 1).

The collaborative efforts of the cerebellum and the hippocampus in tasks with a temporal aspect extend beyond eyeblink conditioning. For example, co-activation between the cerebellum and hippocampus is also reported in a recent fMRI study using a finger tapping task (Onuki et al., 2015). Specifically, co-activation between the cerebellum and hippocampus occurs during a version of the task with a spatio-temporal prediction component, but not during similar tasks when prediction is not required. Similarly, as briefly mentioned earlier, the cerebellum and hippocampus were found to be part of a domain-general pattern separation network, active in both spatial and temporal variations of a delayed match-to-sample task (Paleja et al., 2014).

These studies illustrate the growing support for cerebellar-hippocampal collaborations in healthy physiology. These interactions may also be important in pathophysiology, and we turn now to a recently highlighted example: temporal lobe epilepsy.

## Cerebellar-hippocampal interactions in epilepsy

**Figure 2:**
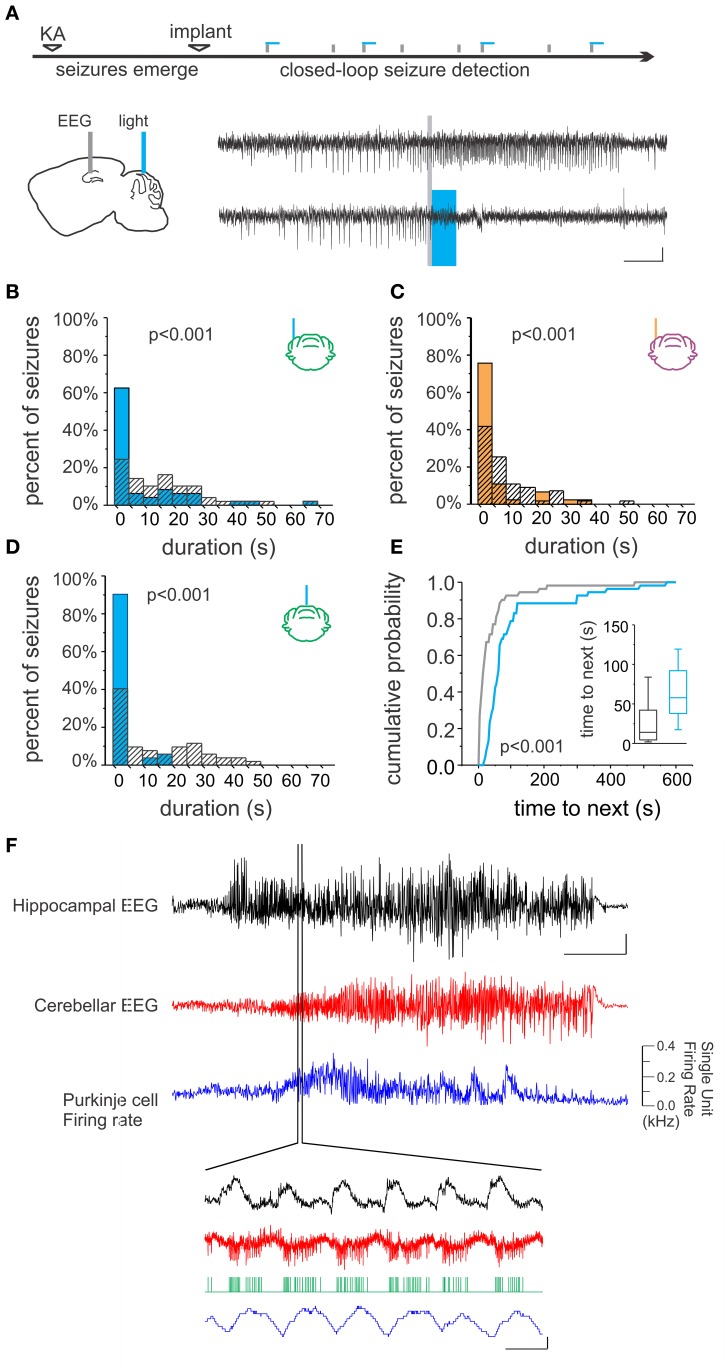
**Bidirectional cerebellar-hippocampal connectivity in epilepsy. (A)** Intrahippocampal kainate injection induced chronic epilepsy and spontaneous seizures. Seizures were detected online, allowing on-demand optogenetic cerebellar modulation selectively at the time of seizures. Top electrophysiological trace is representative of a detected (gray line) seizure recorded from the hippocampus not receiving optogenetic modulation; bottom trace shows attenuation of hippocampal seizure activity resulting from optogenetic cerebellar modulation (horizontal bar indicates light delivery). Stimulation **(B)** or inhibition **(C)** of neurons in the lateral cerebellum or midline vermis **(D)** significantly reduced hippocampal seizure duration. Vermal stimulation uniquely also significantly increased the time to next seizure, indicating a decrease in seizure frequency **(E)**. **(F)** Hippocampal seizure activity (black trace) produced changes in the cerebellar EEG (red trace) and modulated the firing rate of Purkinje cells (blue trace), supporting the bidirectional nature of functional connectivity between the cerebellum and hippocampus. Scale bars: **(A)** 5 s, 0.05 mV; **(F)** Top three traces: 10 s; hippocampal EEG: 1 mV, cerebellar EEG: 0.5 mV. Lower traces: 0.5 mV or 0.1 kHz change in firing rate, 0.1 s. Reproduced with permission from Krook-Magnuson et al. (2014).

The hippocampal formation is the primary focus in mesial temporal lobe epilepsy, which is the most common adult form of epilepsy and also the most drug-resistant (Wiebe, 2000). Though apparently extremely rare, the cerebellum can be the site of origin for seizures in other forms of epilepsy (Harvey et al., 1996; Mesiwala et al., 2002). Studies have revealed a dynamic interplay between the cerebellum and hippocampus during temporal lobe seizures (Figure 2). Remarkably, hippocampal epileptiform activity modulates cerebellar activity, including the firing of juxtacellularly recorded Purkinje neurons (Figure 2F; Mitra and Snider, 1975; Krook-Magnuson et al., 2014). This is true even for spontaneous seizures arising in the chronic stage of the disorder, following an initial insult directly targeting the hippocampus (and not the cerebellum), for example in the intrahippocampal kainate model of temporal lobe epilepsy (Krook-Magnuson et al., 2014; Figure 2F). Importantly, seizures can modify cerebellar activity without, or prior to, generalization; the modification of cerebellar activity does not simply reflect global seizure effects. Additionally, during the secondary generalization of partial seizures originating from the temporal lobe, single photon emission computed tomography (SPECT) indicates that there is a robust increase in blood flow in the cerebellum (Blumenfeld et al., 2009), and after prolonged status epilepticus neuronal cell loss has been observed in both the hippocampus and cerebellum (Tan and Urich, 1984; Soffer et al., 1986; Leifer et al., 1991).

Just as the interactions during information processing appear to be bidirectional, so too are interactions during seizures; not only do temporal lobe seizures alter cerebellar activity, cerebellar activity can also alter temporal lobe seizures. Again, this indicates a unique position of the cerebellum; not all locations within the brain can have an effect on temporal lobe seizures. For example, directly inhibiting granule cells in the dentate gyrus in the hippocampal formation contralateral to the seizure focus does not significantly alter on-going seizures (Krook-Magnuson et al., 2015a). However, while early studies examining electrical stimulation of the cerebellum found promising inhibitory effects on seizures (Cooke and Snider, 1955; Babb et al., 1974; Maiti and Snider, 1975), and clinical trials were initiated (Cooper et al., 1973, 1976), electrical stimulation of the cerebellum was ultimately shown to have mixed effects on seizures (Cooper, 1973; Sramka et al., 1976; Van Buren et al., 1978; Levy and Auchterlonie, 1979; Wright et al., 1984; Davis and Emmonds, 1992; Chkhenkeli et al., 2004; Velasco et al., 2005). Therefore, despite the initial enthusiasm for the cerebellum as a potential target for epilepsy intervention, interest has dropped (for a review, see Fountas et al., 2010).

However, a recent study using more modern techniques with improved specificity of intervention supports renewed interest in this area. Using closed-loop on-demand optogenetics to modulate cerebellar Purkinje cells selectively at the time of seizures, the authors found that hippocampal seizures could indeed be inhibited (Figure 2; Krook-Magnuson et al., 2014). Remarkably, through the use of excitatory and inhibitory light sensitive opsin proteins, it was shown that either excitation (Figure 2B) or inhibition (Figure 2C) of the cerebellum could significantly decrease hippocampal seizure duration. This reduction in seizure duration was found whether the midline (vermis) or the lateral (simplex) cerebellum was targeted with light intervention (Figure 2D). However, stimulation (but not inhibition) of the midline vermis (but not the lateral cerebellum) was capable of also reducing the frequency of spontaneous seizures (Figure 2E). Highlighting the uniqueness of this finding, studies using on-demand optogenetic intervention directly targeting the hippocampus did not find a reduction in seizure frequency (Krook-Magnuson et al., 2013, 2014, 2015a). The promising anti-epileptic results from this study using on-demand optogenetic modulation of the cerebellum support the interpretation that the variable efficacy seen in earlier studies could be due to the lack of temporal and/or cell-type specificity of non-on-demand electrical stimulation.

Importantly, the findings described above suggest that the bidirectional influence between the cerebellum and hippocampus has clinical significance for epilepsy research and treatment. However, just as the circuits involved in the cerebellar-hippocampal interactions discussed in previous sections are still not resolved, more studies are needed to identify the pathways underlying cerebellar-hippocampal interactions in epilepsy. It is important to also note that while cerebellar-hippocampal interactions in temporal lobe epilepsy are striking, they are not necessarily unique. For example, cerebellar neurons can also fire rhythmically and phase-locked with spike and wave discharges in thalamocortical absence epilepsy (Kandel and Buzsáki, 1993), and transgenic mice primarily lacking P/Q-type calcium channel function in Purkinje cells exhibit absence epilepsy (Mark et al., 2011). Additionally, a recent study showed that pharmacological or optogenetic stimulation of the cerebellar nuclei attenuates the generalized spike-and-wave discharges associated with absence epilepsy (Kros et al., 2015). The cerebellum has also been shown to exert influences on neocortical seizures, as electrical stimulation of the cerebellum has shown both beneficial and negative effects on focal seizures of the neocortex (Miller, 1992). As cerebellar effects on seizures are not limited to seizures originating from the hippocampus, a broad mechanism of action beyond simple, direct, hippocampal-cerebellar interactions may be at play (e.g., potentially via brain state regulation).

While closed-loop optogenetic modulation of specific brain areas and circuits could potentially be implemented clinically in the future (Krook-Magnuson and Soltesz, 2015; Krook-Magnuson et al., 2015b), the cerebellum may not be an ideal target for neuroprosthetics. Identifying the mechanism and pathways underlying cerebellar-hippocampal interactions in epilepsy (and the cerebellum in epilepsy more broadly) will therefore provide much needed new potential targets for epilepsy treatment. Additionally, identification of pathways and mechanisms underlying cerebellar-hippocampal interactions may have implications for neurological disorders beyond epilepsy, as the cerebellum has been implicated in a range of disorders, including attention deficit hyperactivity disorder, mood disorders, dyslexia, tinnitus, schizophrenia, and autism spectrum disorders (for a review, see Phillips et al., 2015). For example, fMRI studies indicate reduced cerebellar-hippocampal interactions in schizophrenia (Collin et al., 2011; Duan et al., 2015). While the many potential pathways for the cerebellum and the hippocampus to influence each other presents an initial investigative challenge, they may provide fruitful avenues for intervention in neurological disorders.

## Conclusions

Although, the idea of a broader role for the cerebellum in cognitive functions remains somewhat controversial, there is a growing recognition of its contributions beyond motor learning and control. Fundamentally, recognizing cerebellar-hippocampal interactions means acknowledging the collaborative nature of cognitive processes and appreciating the potential consequences and opportunities this provides. Recent studies establish the importance of cerebellar-hippocampal functional connectivity for spatial and temporal processing and demonstrate the clinical significance of this interaction. However, despite these functional studies, it remains unclear which pathways are critically involved in these interactions. Once elucidated, the specific pathways mediating cerebellar-hippocampal interactions could potentially be targeted for clinical applications.

### Conflict of interest statement

The authors declare that the research was conducted in the absence of any commercial or financial relationships that could be construed as a potential conflict of interest.
